# Leveraging Femtosecond Laser Ablation for Tunable Near-Infrared Optical Properties in MoS_2_-Gold Nanocomposites

**DOI:** 10.3390/nano14231961

**Published:** 2024-12-06

**Authors:** Ilya A. Zavidovskiy, Ilya V. Martynov, Daniil I. Tselikov, Alexander V. Syuy, Anton A. Popov, Sergey M. Novikov, Andrei V. Kabashin, Aleksey V. Arsenin, Gleb I. Tselikov, Valentyn S. Volkov, Alexey D. Bolshakov

**Affiliations:** 1 Moscow Center for Advanced Studies, Kulakova Str. 20, Moscow 123592, Russia; martinov@mitht.org (I.V.M.); ditselikov@mephi.ru (D.I.T.); alsyuy@xpanceo.com (A.V.S.); serjikn@gmail.com (S.M.N.); arsenin@xpanceo.com (A.V.A.); celikov@xpanceo.com (G.I.T.); 2 Laboratory ‘Bionanophotonics’, Institute of Engineering Physics for Biomedicine (PhysBio), National Research Nuclear University MEPhI, Moscow 115409, Russia; aapopov1@mephi.ru; 3 Emerging Technologies Research Center, XPANCEO, Internet City, Emmay Tower, Dubai, United Arab Emirates; vsv@xpanceo.com; 4 CNRS, LP3, Aix-Marseille Université, 13288 Marseille, France; andrei.kabashin@univ-amu.fr; 5 Laboratory of Advanced Functional Materials, Yerevan State University, Yerevan 0025, Armenia; 6 Center for Nanotechnologies, Alferov University, Khlopina 8/3, Saint Petersburg 194021, Russia; 7 Faculty of Physics, St. Petersburg State University, Universitetskaya Emb. 13B, St. Petersburg 199034, Russia

**Keywords:** femtosecond, ablation, molybdenum, disulfide, gold, oxide, nanoparticles, composite, hybrid, core–shell–satellite, near-infrared, photothermal conversion, therapy

## Abstract

Transition metal dichalcogenides (TMDCs), particularly molybdenum disulfide (MoS_2_), have gained significant attention in the field of optoelectronics and photonics due to their unique electronic and optical properties. The integration of TMDCs with plasmonic materials allows to tailor the optical response and offers significant advantages for photonic applications. This study presents a novel approach to synthesize MoS_2_-Au nanocomposites utilizing femtosecond laser ablation in liquid to achieve tunable optical properties in the near-infrared (NIR) region. By adjusting ablation and fragmentation protocols, we successfully synthesize various core–shell and core–shell–satellite nanoparticle composites, such as MoS_2_/MoS_x_O_y_, MoS_x_O_y_/Au, and MoS_2_/MoS_x_O_y_/Au. UV-visible absorption spectroscopy unveils considerable changes in the optical response of the particles depending on the fabrication regime due to structural modifications. Hybrid nanoparticles exhibit enhanced photothermal properties when subjected to NIR-I laser irradiation, demonstrating potential benefits for selective photothermal therapy. Our findings underscore that the engineered nanocomposites not only facilitate green synthesis but also pave the way for tailored therapeutic applications, highlighting their role as promising candidates in the field of nanophotonics and cancer treatment.

## 1. Introduction

Transition metal dichalcogenides (TMDCs) have emerged as a highly promising semiconductor platform due to their unique electronic, optical, and mechanical properties. Among the family of TMDCs, materials such as molybdenum disulfide (MoS_2_) exhibit a direct bandgap in their monolayer form [1], facilitating strong light–matter interactions and enabling applications in optoelectronics, photodetection, and photovoltaics [2,3]. The intrinsic layer-dependent properties of TMDCs offer tunability in electronic and optical characteristics [4,5], making them suitable for a variety of applications ranging from field-effect transistors [6] to flexible and transparent electronics [7].

Novel approaches for the synthesis and fabrication of 2D material-based structures and particles allow us to widen and tailor their properties [8]. The methodologies employed for the acquisition of nanomaterials encompass liquid-phase exfoliation, solvothermal synthesis, and wet chemical synthesis, among others [9,10,11,12]. However, these techniques are accompanied by several limitations, including low yields attributable to extensive purification processes and challenges related to scalability. In turn, laser ablation in liquids (LAL) offers several advantages over traditional nanoparticle (NP) synthesis techniques. LAL features simplicity, robustness, purity of produced NPs/nanostructures, and the elimination of stabilizing reagents or chemical precursors during synthesis. This allows facile green synthesis of colloidal solutions of various NPs and their hybrids.

In particular, early investigations have established the feasibility of TMDC NPs synthesis utilizing femtosecond laser ablation and fragmentation [13,14]. The synthesized TMDC NPs exhibit spherical morphology and preserve the crystalline structure of the parent material. Crucially, they maintain their intrinsic properties, notably a high refractive index and pronounced excitonic characteristics. Moreover, these fabrication methods open new possibilities for synthesizing spherical NPs with a core–shell structure [15]. This core–shell configuration improves the photothermal conversion efficiency (PCE) that features a highly promising methodology for enhancing the precision of oncological interventions—noninvasive photothermal therapy (PTT) [16,17,18]. To selectively target and eradicate malignant cells or neoplasms through the induction of localized hyperthermia, PTT employs photothermal agents that absorb light, subsequently converting it into thermal energy. Recent investigations indicate that both the first (NIR-I, 750–1000 nm) and second (NIR-II, 1000–1700 nm) near-infrared spectral ranges allow for remarkable tissue penetration [19]. Consequently, nanomaterials that exhibit NIR absorption demonstrate significant promise in the context of PTT for disease management and clinical diagnostics [20,21,22].

Despite the previous results demonstrating the high potential of MoS_2_ NPs toward theranostics, bulk MoS_2_ has an indirect bandgap of approximately 1.29 eV, while the monolayer exhibits a direct bandgap of about 1.90 eV [23,24], limiting the absorption in the NIR region. Notably, molybdenum can form various oxides, including MoO_3_, a layered transition metal oxide with a wide bandgap of approximately 3.05 eV [25]. While crystalline MoO_3_ lacks significant absorption in visible and NIR spectral ranges, defects in MoO_3−x_ enhance its NIR absorption due to changes in electronic structure and plasmonic effects, making it a promising photothermal agent for PTT [26,27]. Molybdenum-based nanomaterials possess three key appealing properties: (1) they exhibit excellent biocompatibility and low long-term cytotoxicity, while molybdenum accumulation does not significantly threaten patient health [28,29]; (2) their high surface-to-volume ratios enable them to act as drug delivery platforms for various chemotherapeutic agents, enhancing chemotherapy while minimizing side effects [30,31]; and (3) they have strong absorption in the NIR window, allowing for efficient conversion of light energy into heat for effective photothermal therapy [32,33,34,35].

Metal-based plasmonic nanocomposites integrate multiple features to enhance the efficiency of therapy and diagnostics [36]. So, the integration of MoS_2_ with noble metal NPs, such as gold (Au), can enhance charge carrier dynamics and enable surface plasmonic effects, providing avenues for improved performance. For instance, in [37], the authors demonstrated synergetic MoS_2_/Au action leading to active peroxidase mimetic activity allowing for enhanced sensing. Improved catalytic behavior of Au-MoS_2_ quantum dot composites was reported in [38]. Highly sensitive plasmonic-driven detection of NO_2_ was obtained with MoTe_2_ NPs [39]. As such, the hybrid NPs can exhibit unique optical, electronic, and catalytic properties, driven by the synergistic effects arising from the TMDC-metal interactions. The ability to fine-tune the synthesis parameters during laser ablation makes it an attractive approach for developing advanced nanocomposites with specific functionalities.

In this study, we leverage the advantages of LAL to synthesize MoS_2_/Au composite particles with a unique core–shell–satellite architecture. By varying our synthesis protocol across different approaches—namely, ablation, fabrication, and mixture—we explore the effects of the fabrication protocol on the structural and optical properties of the resulting NPs. These composite particles are meticulously characterized using electron microscopy, Raman spectroscopy, energy-dispersive X-ray spectroscopy, and optical absorption spectroscopy, providing insight into their morphology and interaction with light. Our optical characterization results reveal a pronounced ability to tailor the NIR-I response as a function of the chemical composition, showcasing the potential for achieving optimized photothermal conversion efficiencies. This tunability not only enhances the efficiency of PTT but also paves the way for the development of promising theranostic applications. By harnessing the synergistic effects of the MoS_2_ and Au components, our composite NPs hold great potential for targeted disease management, combining both therapeutic and diagnostic functionalities in a single platform.

## 2. Results and Discussion

### 2.1. Fabrication and Characterization of Nanoparticles

The synthesis of NP solutions is carried out by the method of femtosecond LAL. First, plasma is generated due to the intense heating of a target with a laser. Second, solid target material transforms into plasma “evaporated” in liquid. Third, NPs nucleation takes place in the cavitation nanobubbles which trap plasma [40,41]. In brief, rapid and localized heating provided by the femtosecond laser pulses leads to high nucleation rates and short reaction times, facilitating the efficient synthesis of NPs.

**Figure 1:**
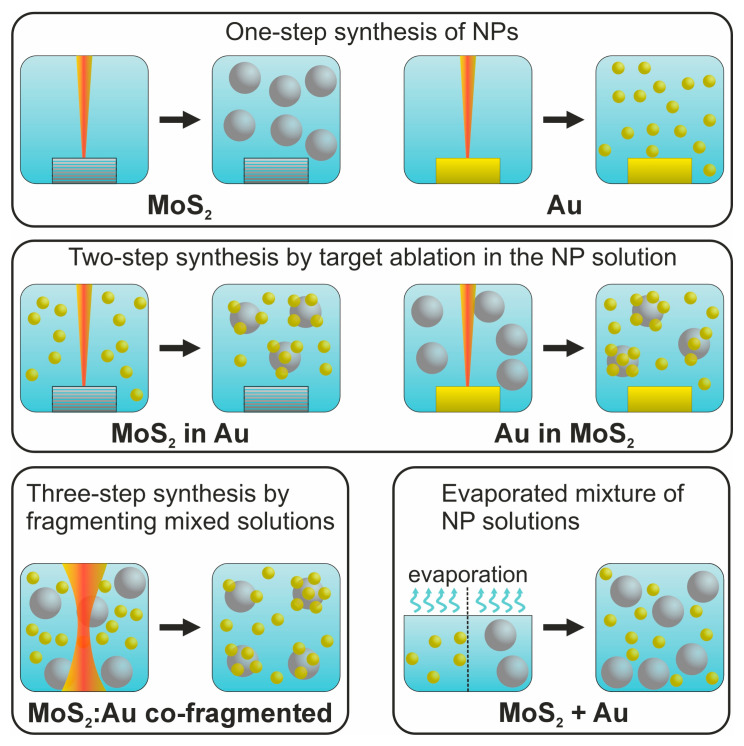
Schematic representation of the one-, two-, and three-step processes as well as solution mixing for the synthesis of pristine and hybrid MoS_2_/Au NPs.

The samples obtained by a conventional one-step femtosecond laser ablation of MoS_2_ and Au targets are denoted as “MoS_2_” and “Au” (see top schematic in Figure 1). Additionally, we combined various synthesis routes to obtain hybrid (composite) NPs. First, we conducted a two-step synthesis by ablating the MoS_2_ (or Au) targets in pre-synthesized colloidal solutions of Au (or MoS_2_) NPs, respectively (see schematic in Figure 1). The obtained composite NPs are designated as “MoS_2_ in Au” and “Au in MoS_2_”, correspondingly.

Additionally, we performed a three-step synthesis by co-fragmenting two colloidal solutions (of MoS_2_ and Au NPs) mixed in one pot taken at a 1:1 volume ratio. This sample is referred to as “MoS_2_:Au co-fragmented” (see schematic in Figure 1). For the straightforward characterization of the NP composites, we prepared a reference sample—a simple mixture of the two pristine NP solutions in a 1:1 ratio without their co-fragmentation. This sample is denoted as “MoS_2_+Au” (see the last schematic in Figure 1).

### 2.2. Morphology and Chemical Composition

**Figure 2:**
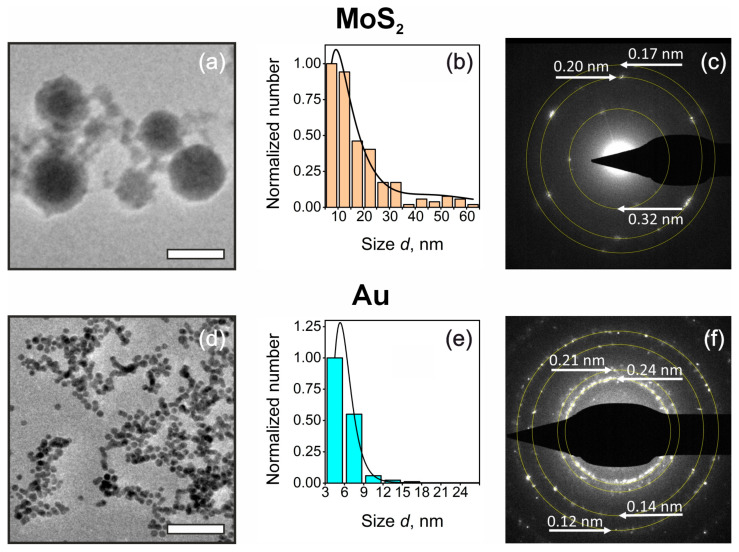
TEM characterization of one-step synthesized NPs. (**a**,**d**) TEM images of the ablated MoS_2_ (**a**) and Au (**d**) NPs. Scale bar, 50 nm. (**b**,**e**) Size distributions of MoS_2_ (**b**) and Au (**e**) NPs. (**c**,**f**) SAED patterns of MoS_2_ (**c**) and Au (**f**) NPs.

We start with a characterization of pristine ablated NPs. Figure 2a,d shows typical TEM images of the NPs, and their size distributions are depicted in Figure 2b,e. According to the latter, MoS_2_ NPs demonstrate bimodal size distribution with two characteristic mean diameters <d_1_> = 18 nm and <d_2_> = 50 nm. These values are in good agreement with previously reported data [13,14] and can be explained by the influence of the cavitation bubble on the formation of the NPs during laser ablation. At the same time, the size of Au NPs appeared to be significantly smaller (<d_Au_> = 5.9 nm). The reduction in NP size is assumed to be the result of the addition of 1 mM of NaCl to the solution for the ablation of the Au target. The variation of NaCl content as well as changing the pH of the solvent are well-known tools for tuning the average size of laser-ablated Au NPs [42]. Another notable aspect is the presence of large core–shell NPs in the MoS_2_ sample seen in Figure 2a. Their structure will be assessed in more detail below.

Based on selected area electron diffraction (SAED) data (Figure 2c), MoS_2_ NPs exhibit a pronounced degree of single-crystal characteristics. The SAED pattern shows 2H MoS_2_ reflections with corresponding 3.2 Å interplanar spacing, which aligns with the hexagonal MoS_2_ lattice parameter a = 3.18 Å [43,44,45]. Furthermore, minor reflexes corresponding to the lattice parameter d = 2.02 Å and 1.73 Å are associated with the crystalline structure of h-MoO_3_ (320) [46] and m-MoO_2_ ($11\bar{3}$) [47] oxides [48], respectively. The presence of SAED patterns related to MoS_2_, MoO_2_, and MoO_3_ indicates the partial oxidation of the initial MoS_2_ during the LAL [14].

SAED pattern in Figure 2f reveals that the gold NPs display a diffraction ring together with intense reflexes, which indicates their polycrystalline characteristics in contrast to MoS_2_ NPs. The observation of multiple diffraction rings suggests a heterogeneous orientation of crystallites within the specimen. The evaluated interplanar spacings d = 2.42 Å (111), 2.10 Å (200), 1.14 Å (220), and 1.23 (311) Å correspond well to the lattice of gold (hkl denoted in brackets assigned via JCPD-ICDD 00-004-0784 card).

**Figure 3:**
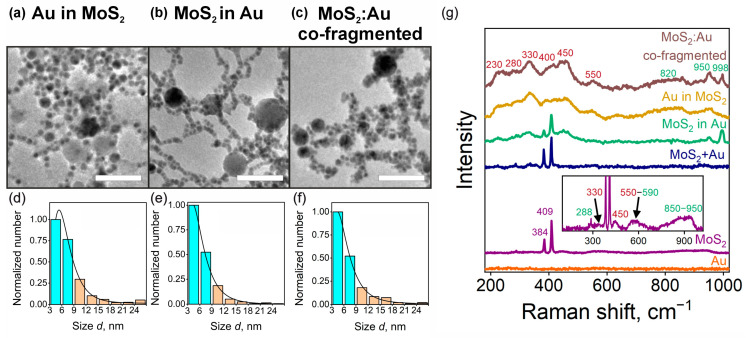
Characterization of two- and three-step synthesized NPs and Raman spectroscopy data. TEM images of (**a**) “Au in MoS_2_”, (**b**)“MoS_2_ in Au”, and (**c**) “MoS_2_:Au co-fragmented” NPs. Scale bar, 50 nm. Size distributions of (**d**) “Au in MoS_2_”, (**e**) “MoS_2_ in Au”, and (**f**) “MoS_2_:Au co-fragmented” NPs. Turquoise bars represent Au NPs, while orange bars represent MoS_2_-based NPs. (**g**) Raman spectra of the NPs. Violet, brown- and green-colored numbers indicate the positions of the peaks related to MoS_2_, MoS_x_O_y_, and MoO_x_, respectively. Inset shows the magnified low-intensity peaks of the MoS_2_ sample.

Next, we analyze the composite NPs. Figure 3a–f depict transmission electron microscopy (TEM) images and size distributions of the NPs synthesized via two- and three-step processes. The mean diameter of “Au in MoS_2_”, “MoS_2_ in Au”, and “MoS_2_:Au co-fragmented” NPs resemble those of the pristine Au samples, although they exhibit a significantly broader dispersion. Despite the observation of larger MoS_2_ NPs in the TEM images, the size distributions of the composites differ markedly from that of the pristine MoS_2_ samples. This observation suggests that the number of smaller Au NPs substantially surpasses that of larger MoS_2_-rich NPs produced during equal ablation periods. To elucidate these findings, it is important to highlight that Au NPs are not susceptible to laser irradiation, as evidenced by the higher ablation threshold of gold (0.4 J·cm^−2^) compared to that of molybdenum disulfide, which has an ablation threshold of 0.15 J·cm^−2^ [49,50]. This distinction allows us to identify particles with diameters below 9 nm as Au NPs, while those exceeding 9 nm are classified as MoS_2_, as indicated by the histogram color coding in Figure 3d–f.

In Figure 3g, we present typical Raman spectra of the ablated pristine Au, MoS_2_, and hybrid Au/MoS_2_ structures. Spectra are background-subtracted and normalized to similar intensities. As expected, Au NPs demonstrate no significant Raman response. The spectrum of ablated MoS_2_ demonstrates a set of lines typical for crystalline MoS_2_, such as 384 cm^−1^ (E_2g_ mode) and 409 cm^−1^ (A_1g_ mode) [51,52], indicating their crystallinity. Apart from these lines, the inset in Figure 3g demonstrates magnified low-intensity peaks at 330, 450, and 550 cm^−1^ indicative of the MoS_x_O_y_ ternary compound formation [53]. This phenomenon will be discussed in more detail hereafter for the hybrid MoS_2_/Au NPs. The low-intensity narrow peak at 288 cm^−1^ and the plateau at 850–950 cm^−1^ are related to MoO_3_ [54], while the 590 cm^−1^ peak relates to MoO_2_ oxide [55]. Thus, the Raman spectroscopy data of the pristine MoS_2_ sample are fully in agreement with the SAED results showing the presence of crystalline MoS_2_ and oxide formation during the ablation. The spectra of the “MoS_2_+Au” (mixture of pristine NPs) do not exhibit a considerable difference from the MoS_2_ sample. The latter demonstrates that concentrating the solutions by partial evaporation of the liquid media followed by their mixture does not affect the NPs.

Apart from MoS_2_-related lines, the “MoS_2_ in Au” sample demonstrates wide bands at 230, 280, 330, 400, and 450 cm^−1^, as well as narrow lines at 950 and 998 cm^−1^. These peaks can be interpreted as follows: 230 cm^−1^ is attributed to Mo–Mo bonds, the 280 cm^−1^ line can be assigned to Mo–S vibrations, 330 is related to Mo–O bonding, while the 400 and 450 cm^−1^ lines are ascribed to S_apical_-Mo vibrations (here, the “apical” index refers to the vibrations of atoms located at the edges, i.e., apexes, of a structure) [53,56]. The line at 550 cm^−1^ is typical for S-S bonds [57]. Overall, the three-peak feature with the bands in the vicinity of ~200 cm^−1^, ~300 cm^−1^, and ~450 cm^−1^ resembles the Raman spectra of MoS_x_O_y_ structures [53]. The observation of a peak at 998 cm^−1^ provides strong evidence for the formation of crystalline MoO_3_ [58]. Furthermore, 820 and 950 cm^−1^ are distinctive peaks of amorphous MoO_3_ [59]. The spectra of “Au in MoS_2_” and the “MoS_2_:Au co-fragmented” sample exhibit similar broad bands, while the lines indicative of crystalline MoS_2_ are absent in these samples. This result shows that, unlike Au NPs, MoS_2_ NPs are considerably oxidized and amorphized under fragmentation.

In summary, the Raman spectra of MoS_2_ ablated in water demonstrate intense lines attributed to hexagonal MoS_2_ as well as low-intense oxide and sulfoxide bands. MoS_2_ ablation in Au solution (“MoS_2_ in Au” samples) allows the retention of MoS_2_ structure, although the fractions of both MoS_x_O_y_ and MoO_3_ phases increase in comparison to one-step-synthesized MoS_2_ NPs. Raman spectroscopy reveals that laser interaction with MoS_2_ NPs in an aqueous solution affects their crystallinity and leads to the formation of both oxides and sulfoxides.

### 2.3. Composition and Structure of Composite Nanoparticles

**Figure 4:**
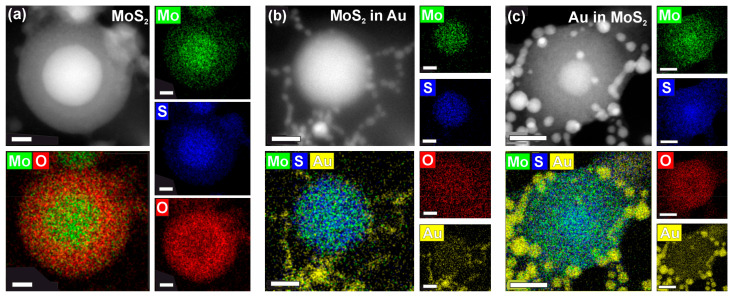
High-angle annular dark-field imaging (upper left panels) and EDX maps of “MoS_2_” (**a**), “MoS_2_ in Au” (**b**), and “Au in MoS_2_” (**c**) NPs. Scale bar, 20 nm.

In order to investigate the hybrid composites in detail, we performed high-angle annular dark-field (HAADF) studies and energy-dispersive X-ray spectroscopy (EDX) mapping of the NPs. The HAADF image of the ablated MoS_2_ (Figure 4a) shows its core–shell structure. The EDX maps reveal the Mo- and S-rich core with a radius of 25 nm and a 20 nm thick oxygen-rich shell. These results are in agreement with Raman and SAED studies. Noteworthy, oxidation of the shell allows us to fine-tune the optical properties of the NPs, which will be discussed in detail in the following sections.

Both composite NPs of “MoS_2_ in Au” (Figure 4b) and “Au in MoS_2_” (Figure 4c) samples show that Au NPs are positioned as “satellites” of the MoS_2_-rich cores. Though it is hard to interpret whether the Au NPs are integrated into the core in “MoS_2_ in Au” NPs, it is evident from Figure 4c that small Au particles cover the bigger oxidized NP. As to the core structure, “MoS_2_ in Au” NPs exhibit an oxygen-free core and can be considered MoS_2_ core/Au satellite NPs, which proves that MoS_2_ retains crystallinity when ablated in the solution of Au NPs. At the same time, MoS_2_ NPs fragmented throughout the second step of “Au in MoS_2_” synthesis exhibit only a small MoS_2_-rich core covered with a thick oxygen-rich MoS_x_O_y_ shell. This again confirms that MoS_2_ is considerably oxidized during the fragmentation and reveals that the synthesized NPs possess the structure of “MoS_2_ core/MoS_x_O_y_ shell/Au satellite”.

### 2.4. Optical Properties and Photoheating

**Figure 5:**
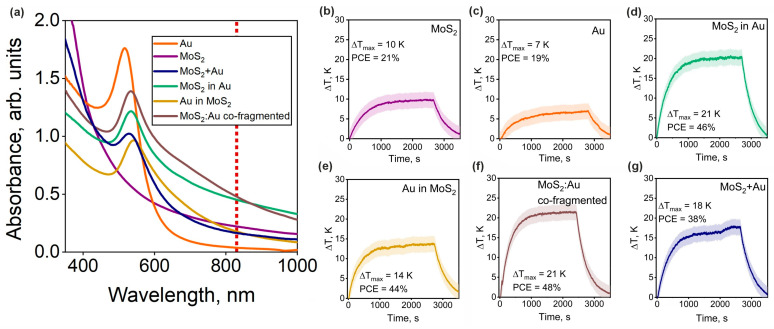
Optical absorption and photoheating. (**a**) UV-visible extinction spectra. Red dotted line indicates the photoheating laser wavelength. (**b**–**g**) Photoheating dynamics. ΔT_max_ and PCE notations indicate the values of maximum temperature increases observed throughout the heating and photothermal conversion efficiencies. The plots are presented in the following order: (**b**) MoS2, (**c**) Au, (**d**) MoS_2_ in Au, (**e**) Au in MoS_2_, (**f**) MoS_2_:Au co-fragmented, (**g**) MoS_2_+Au.

To investigate the optical properties of the synthesized NPs, UV-Vis spectroscopy was employed. The analysis of absorbance spectra and the identification of peculiarities indicative of interactions between MoS_2_ and Au allow us to explore additional degrees of freedom for the optical tuning of the composites. Figure 5a shows the absorbance spectra of pristine NPs and all the fabricated hybrids. Pristine Au NPs exhibit a characteristic surface plasmon resonance peak at 520 nm typical for spherical Au [60]. Pristine MoS_2_ NPs exhibit monotonous decay of the absorbance without prominent spectral features. Importantly, despite the absence of resonant absorption, MoS_2_ NPs show much more intense absorption compared to Au NPs for the wavelength exceeding 600 nm.

All the composite NPs display distinct plasmon excitation around 540 nm, manifesting the interaction between the two types of NPs that results in a red shift of Au plasmon resonance. This effect is ascribed to electron spill-out due to the interaction with surrounding MoS2/MoS_x_O_y_ media [61]. It is also worth noting that “MoS_2_:Au co-fragmented” and “MoS_2_ in Au” spectra exhibit the appearance of a 650–750 nm shoulder. This phenomenon is assumed to be associated with the formation of defect levels in molybdenum oxides and sulfoxides [62]. Previously, we observed a similar effect with MoS_2_ NPs synthesized in ethanol [14]. Thus, among the obtained hybrids, the “MoS_2_ in Au” and “MoS_2_:Au co-fragmented” samples are assumed to be the most promising for biomedical use since they exhibit the highest extinction coefficients in the NIR-I range (750–900 nm) among the considered materials.

In order to evaluate the applicability of the synthesized composites for photothermal therapy, we carried out a series of experiments aimed at assessing their photothermal conversion efficiency. For this, we precisely monitored the temperature change in the synthesized colloidal solutions under continuous wave illumination provided by a laser diode operating at a wavelength of 830 nm.

As can be seen from the thermograms (Figure 5b–g), the lowest PCE as well as the increase in temperature (Δ*T_max_*) was shown by pristine Au and MoS_2_ (one-step synthesized), with PCE values of 19% and 21%, respectively. The best performance among the hybrids was shown by “MoS_2_:Au co-fragmented” and “MoS_2_ in Au” NPs exhibiting 48% and 46% PCE, respectively. These results correlate well with the absorbance data in Figure 5a. To confirm the effect of the mutual Au-MoS_2_ influence and specific optical performance of the core–satellite morphology through surface modification with gold NPs, we measured a thermogram of the MoS_2_+Au mixture at the same concentration. This sample showed a significantly lower PCE of 38%. The observed temperature change and the efficiency of light conversion are the additive sum of the corresponding characteristics of the two initial solutions of pristine Au and MoS_2_ NPs.

## 3. Conclusions

This investigation reveals a novel approach for synthesizing Au/MoS_2_-based composite NPs via femtosecond LAL. Utilizing one-, two-, and three-step synthesis methodologies, we successfully fabricated MoS_2_/MoS_x_O_y_, MoS_x_O_y_/Au, and MoS_2_/MoS_x_O_y_/Au hybrids exhibiting core–shell and core–shell–satellite configurations. The emergence of MoS_x_O_y_ is attributed to the inherent susceptibility of MoS_2_ to oxidation under laser irradiation. The presence of defects within molybdenum oxides and sulfoxides significantly enhances the NPs’ absorption in the NIR range.

Our findings underscore that the femtosecond laser ablation technique not only facilitates an eco-friendly synthesis of Au/MoS_2_-based hybrids but also enables the precise tuning of the optical properties of these nanocomposites. Remarkably, the optimal configurations, specifically “MoS_2_ in Au” and “MoS_2_:Au co-fragmented”, achieved PCE exceeding 46% under 830 nm laser excitation within the NIR-I window, underscoring their substantial potential for selective photothermal therapy. The distinctive PCE values and adjustable absorption characteristics render MoS_2_/MoS_x_O_y_/Au hybrids as promising candidates for the advancement of nanophotonic applications and targeted therapeutic strategies in cancer treatment.

## 4. Materials and Methods

### 4.1. Materials

The MoS_2_ target was purchased from 2D Semiconductors Inc. (Scottsdale, AZ, USA). The gold target of 99.999% purity was purchased from Girmet (Moscow, Russia). All solutions were prepared in ultrapure water with 18.3 MOhm·cm resistivity.

### 4.2. Synthesis of Single-Component and Hybrid NPs

The setup for the synthesis of NPs consists of the following components: A Yb:KGW laser system (1030 nm, 250 fs, 25 µJ, 200 kHz, TETA-20 model, Avesta, Moscow, Russia); the crystalline target of the material was placed in a glass cuvette (BK-7, wall thickness 3 mm) fixed vertically in a chamber; the cuvette was filled with 20 mL of distilled water; the laser beam was focused on the target surface using an F-Theta lens (100 mm focal distance, Thorlabs, Newton, NJ, USA) installed in the galvanoscanner (LScan-10, Ateko-TM, Moscow, Russia); the device itself is designed to evenly distribute laser radiation on the target surface, thus increasing the productivity of synthesis. To estimate an average laser fluence at the target surface, we measured the size of the ablation crater after a single laser pulse (Figure S1). The measured spot size was 87 μm, with a corresponding average laser fluence of 0.1 J/cm^2^. Note that we report a mean laser fluence for the ablation, which is different from the conventionally reported peak fluence value [63]. This is due to a strong self-focusing of femtosecond laser pulses in liquids [64], which makes standard spot size characterization techniques generally used for picosecond laser ablation unapplicable [65].

### 4.3. One-Step Synthesis of “MoS_2_” and “Au”

Pristine NPs were fabricated by the femtosecond LAL method. The synthesis time was 40 min for each sample. As a result, we obtained two solutions of dark brown and deep red colors for MoS_2_ and Au, respectively. To obtain a fine fraction of gold NPs, the solution was additionally separated using a centrifuge (Centrifuge 5425 R, Eppendorf, Hamburg, Germany). The centrifuge speed was set to 15,000× *g* and the operating time was 15 min. After this procedure, the supernatant with the required fraction of NPs was collected.

### 4.4. Two-Step Synthesis of “MoS_2_ in Au” (and “Au in MoS_2_”) Hybrids

The crystalline targets of MoS_2_ and Au, respectively, were placed in the pre-synthesized colloidal solution of Au and MoS_2_ NPs at concentrations of 0.3 g·L^−1^. Thus, the ablation of the targets was performed in a solution of the pre-synthesized NPs under the same conditions as the one-step synthesis.

### 4.5. Three-Step Synthesis “MoS_2_:Au Co-Fragmented”

To synthesize the composites, two colloidal solutions (MoS_2_ and Au) were taken at concentrations of 0.3 g·L^−1^. The solutions were mixed in a 1:1 volume ratio. After this procedure, the solution mixtures were fragmented together in the same glass cuvette. The fragmentation geometry was in the transmission of the solution until the supercontinuum of white light was reached, and the synthesis time was 7 min.

### 4.6. Reference Mixture of the Two Pristine NP Solutions (“MoS_2_+Au”)

To obtain this sample, half of the solvent in both “MoS_2_” and “Au” solutions was evaporated prior to the mixing to ensure that the mass concentration of each component corresponded to the ones produced by the two- and three-step approaches.

### 4.7. Characterization of the NPs

The concentration was estimated gravimetrically by measuring the mass of the dry portion of the solution of a fixed volume of the solution on a high-precision scale (±0.01 mg).

### 4.8. Electron Microscopy and Sample Preparation

Samples visualization was implemented by TEM with a JEOL JEM-2100 microscope (Tokyo, Japan). For TEM studies, 20 μL of the solutions were drop-cast onto the carbon-coated copper TEM grids and dried for 30–40 min in ambient conditions. To obtain the size distributions, we processed the TEM images via Gwyddion software (version 2.65) [66]. To distinguish the NPs, we used Otsu’s threshold selection method built in Gwyddion software (version 2.65) [67]. Size distribution histograms, which were obtained from the processing of the arrays of doubled radii of equivalent discs, were fitted with log-normal distributions. Only the NPs with sizes exceeding 3 nm were taken into account. Each distribution considered 180–680 NPs.

HAADF and EDX mappings were obtained on Titan Themis Z TEM (ThermoFisherScientific, Eindhoven, The Netherlands) with a Super-X EDX detector.

### 4.9. Raman Spectroscopy

Raman spectra were acquired with the Horiba LabRAM HR Evolution (HORIBA Ltd., Kyoto, Japan) confocal Raman microscope. The excitation wavelength was 633 nm. 100×/N.A. = 0.90 microscope objective and 600 lines/mm diffraction grating were used. Spectra were obtained from the NPs deposited on cover glasses by drop-casting. An incident laser power of 0.1 mW was used for the studies to avoid sample degradation. The acquisition time of each point was 10 × 30 s. Five spectra were obtained for each sample and the most typical one was analyzed. Only a slight variation of the spectra at various points was observed.

### 4.10. Absorbance Spectroscopy

The optical extinction spectra were analyzed using a spectrophotometry method with the MC 122 system (SOL Instruments, Minsk, Belarus) in the spectral range of 330–1000 nm. For the measurements, glass cuvettes with a 10 mm optical path length filled with 1 mL of colloidal solution were used.

### 4.11. Photothermal Measurement

A laser emitting at a wavelength of 830 nm equipped with a reflector collimator was used to produce a collimated beam with an approximate diameter of 2 mm. The emitted radiation passed through a standard UV–Vis quartz cuvette (with a 1 cm optical path length) containing 1 mL of the NP suspension at a concentration of 0.2 mg·mL^−1^. Additionally, a thermal power meter (Thorlabs PM100D) was positioned behind the sample to quantify the light power both prior to and after the introduction of the sample. We ensured that the illumination power of 0.7 W was within the safety recommendations for photothermal treatment. On–off experiments (40 + 20 min) were performed; a duration of 40 min was selected as the optimal interval to minimize sample evaporation while attaining thermal saturation. An infrared thermal imaging camera (FLIR C3, FLIR Systems, Wilsonville, OR, USA) was positioned above the cuvette to observe the thermal dynamics and assess the temperature.

### 4.12. The Photothermal Conversion Efficiency (PCE)

PCE was calculated using the equation:

$$PCE=\frac{\Delta T_{max}m_{solution}C_{solution}B}{\left(I_{0}-I_{tr}\right)}*100\%,$$

where $\Delta T_{max}$ is the maximum temperature difference between the studied NP solution and the ambient temperature, $m_{solution}$ is the mass of the solvent, $C_{solution}$ is the specific heat capacity of the solvent, *B* is the time constant defined as the slope of the cooling time from the logarithm of the quantity ($\frac{\Delta T_{max}}{\Delta T(t)})$, $I_{0}$ is the laser radiation power, and $I_{tr}$ is the laser radiation power after passing through the NP solution [68].

## Data Availability

Data are contained within the article and Supplementary Materials.

## Supplementary Materials

The following supporting information can be downloaded at: https://www.mdpi.com/article/10.3390/nano14231961/s1, Figure S1. SEM image of a single-shot ablation crater. Yellow circle defines the irradiated area with 43.46 μm radius.
