# Supplementary material for: Leveraging Femtosecond Laser Ablation for Tunable Near-Infrared Optical Properties in MoS2-Gold Nanocomposites
Source: Nanomaterials (Basel). 2024 Dec 6;14(23):1961. doi: 10.3390/nano14231961 (PMC11643533; doi:10.3390/nano14231961)
Supplement: Supplementary file 1 [file nanomaterials-14-01961-s001.zip › nanomaterials-3347488-supplementary.pdf]

Supplementary information

Leveraging Femtosecond Laser Ablation for Tunable Near-Infrared Optical Properties in MoS2-Gold Nanocomposites

Ilya A. Zavidovskiy [1,*], Ilya V. Martynov [1], Daniil I. Tselikov [1,2], Alexander V. Syuy [1,3], Anton A. Popov [2], Sergey M. Novikov [1], Andrei V. Kabashin [4], Aleksey V. Arsenin [1,3,5], Gleb I. Tselikov [1,3], Valentyn S. Volkov [3] and Alexey D. Bolshakov [1,5,6,7,*]

[1] Moscow Center for Advanced Studies, Kulakova str. 20, Moscow, 123592, Russia; ilyazavid@yandex.ru (I.A.Z); martinov@mitht.org (I.V.M.); ditselikov@gmail.com (D.I.T.); alsyuy@xpanceo.com (A.V.S.); arsenin@xpanceo.com (A.V.A.); celikov@xpanceo.com (G.I.T.); serjikn@gmail.com (S.M.N.); bolshakov@live.com (A.D.B.)

[2] Laboratory 'Bionanophotonics', Institute of Engineering Physics for Biomedicine (PhysBio), MEPhI, Moscow 115409, Russia; aapopov1@mephi.ru (A.A.P.)

[3] Emerging Technologies Research Center, XPANCEO, Internet City, Emmay Tower, Dubai, 000000, United Arab Emirates; vsv@xpanceo.com (V.S.V.)

[4] CNRS, LP3, Aix-Marseille Université, Marseille 13288, France; andrei.kabashin@univ-amu.fr (A.V.K.)

[5] Laboratory of Advanced Functional Materials, Yerevan State University, Yerevan, 0025, Armenia

[6] Alferov University, Khlopina 8/3, Saint Petersburg 194021, Russia

[7] Faculty of Physics, St. Petersburg State University, Universitetskaya Emb. 13B, St. Petersburg 199034, Russia

* Correspondence: ilyazavid@yandex.ru (I.A.Z); bolshakov@live.com (A.D.B.).

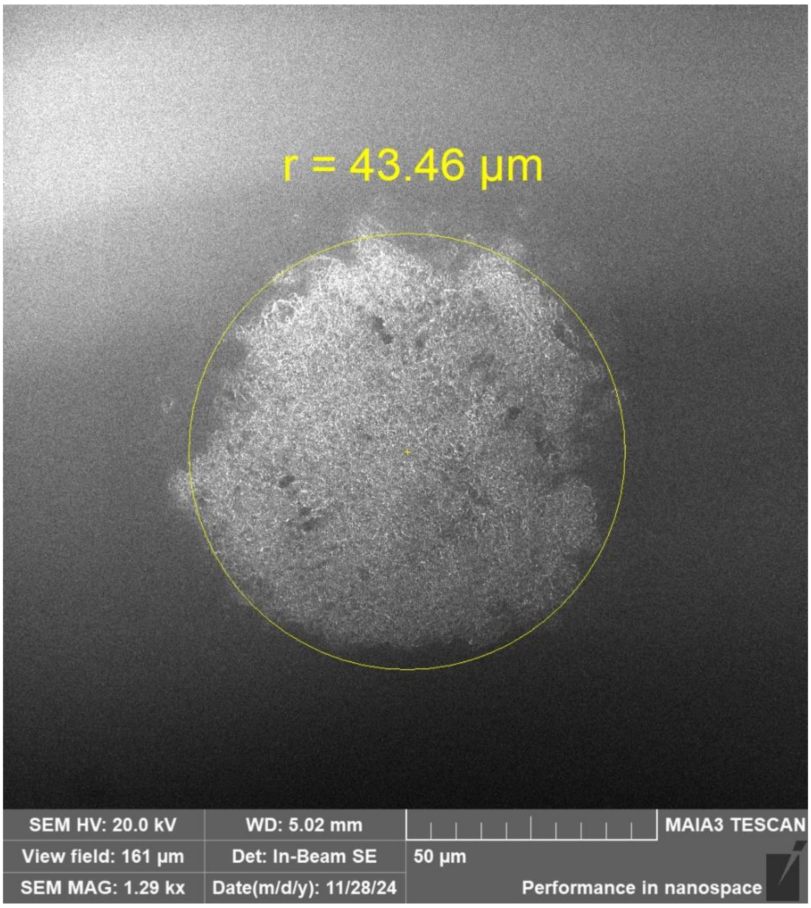

Figure S1. SEM image of a single-shot ablation crater. Yellow circle defines the irradiated area with 43.46 µm radius.
